# Added Value of Nucleic Acid Testing in Blood Banks: A 15-Year Retrospective Study from India

**DOI:** 10.7759/cureus.98684

**Published:** 2025-12-08

**Authors:** Sangeeta Pathak, Surjit Singh, Tamojit Chakraborty, Satish Kaushik, Ruchi Dubey

**Affiliations:** 1 Transfusion Medicine, Max Super Speciality Hospital, New Delhi, IND; 2 Laboratory, Max Super Speciality Hospital, New Delhi, IND

**Keywords:** nucleic acid test, replacement donors, serology, transfusion safety, transfusion-transmitted infections, voluntary donors

## Abstract

Background

Viral transfusion-transmissible infections (TTIs), hepatitis B (HBV), hepatitis C (HCV), and human immunodeficiency virus (HIV) pose a major risk to blood safety in India, where most blood banks rely on serology alone for TTI screening. Our blood bank at Max Healthcare, New Delhi, India, has used nucleic acid testing (NAT) alongside serology for TTI screening since 2010.

Objectives

The aim of this study was to evaluate 15 years of real-world blood bank data to evaluate the impact of NAT alongside serology in improving TTI detection and transfusion safety.

Methods

This single-centre retrospective observational study analysed blood donor, serology, and NAT data from 211,555 donations between January 2010 and December 2024. All donations underwent chemiluminescence immunoassay (CLIA)-based serology for TTI detection. All seronegative samples were subject to NAT on the Cobas MPX polymerase chain reaction (PCR)-based minipool NAT (Roche, Basel, Switzerland). Seropositive donations and NAT-reactive donations were discarded.

Results

A total of 2,04,609 (96.72%) were replacement donations. A total of 3,333 (1.58%) donations were seropositive for viral TTIs, and most of these were from replacement donors (N=3,289; 98.68%). Seropositivity was highest for HBV (N=1,460; 43.78%), followed by HCV (N=1,213; 36.39%) and HIV (N=660; 19.8%). NAT detected 205 additional TTI cases missed by serology; most (N=164; 79.76%) were HBV NAT yields, followed by HCV (N=35; 16.83%) and HIV (N=7; 3.41%). There was one case of HBV-HCV co-infection. The overall NAT yield rate was 1:1005, and the HBV NAT yield rate was 1:1262.

Conclusion

NAT enhances blood safety by detecting infections missed by serology, supporting the need for mandatory nationwide implementation despite cost and infrastructure challenges.

## Introduction

Transfusion-transmissible infections (TTIs), particularly the viral TTIs caused by the hepatitis B virus (HBV), hepatitis C virus (HCV), and human immunodeficiency virus (HIV), remain a major barrier to blood safety in India. Among Indian blood donors, recent seroprevalence estimates range from 2% to 8% for HBV, 0.5% to 1% for HCV, and 0.16% to 0.30% for HIV [1]. Blood safety regulations in India are governed by the National Blood Transfusion Council (NBTC) and the National AIDS Control Organization (NACO). The 2017 NBTC/NACO guidelines mandate the screening of these three viral TTIs, along with malaria and syphilis, using ‘a test of high sensitivity’ without specifying the type of test, and recommend that all samples with initial non-reactive results be considered free of infection [2]. Consequently, most blood banks in India rely on serological screening via enzyme-linked immunosorbent assays (ELISA) or chemiluminescent microparticle immunoassays (CLIA) for TTI screening. Despite this, the risk of TTI transmission from recently infected but seronegative donors persists because serology fails to detect infections during the early window period (WP) before antibodies develop. Since replacement donors continue to outnumber voluntary donors in India [3,4], stringent donor selection might not be practical for all donations. As a result, having an additional layer of blood safety beyond serology becomes essential.

Nucleic acid testing (NAT) directly detects circulating viral nucleic acids in blood samples without depending on the body’s immune response, unlike serology: as a result, NAT has a higher sensitivity for TTI detection compared to serology [5]. When used along with serology, NAT considerably lowers the WP of TTI detection, thereby enhancing transfusion safety [5,6]. NAT can be performed on individual donations (ID-NAT) or on minipools (MP-NAT) containing samples from a small group of donors [7]. First introduced in the 1990s, NAT is now a mandatory screening modality for detecting viral TTIs in donated blood in developed countries [8]. Even in India, despite being associated with a relatively higher cost for installation, training, and reagents, several experts have called for routine usage of NAT in addition to serology for TTI screening to improve blood safety in India [1,6,9]. However, as of this writing, NAT has not been made mandatory in Indian blood banks.

NAT was first made available in India in 2008 and has since been implemented in select blood banks, particularly in tertiary care centres and private institutions, though its uptake remains inconsistent [10]. Our centre was one of the earlier adopters of NAT: starting from 2010, we have been using the Roche Cobas platform (Basel, Switzerland) to routinely perform MP-NAT for detecting viral TTIs in seronegative samples. Over the past 15 years, we have collected data relating to serological tests and NAT in our blood bank. The present study was planned with the objective of compiling our blood bank data to explore transfusion and TTI trends in our region and also to evaluate the value of routine NAT usage in addition to serology.

## Materials and methods

Study setting, study participants

This retrospective observational study involved compilation and analysis of data routinely collected at the blood bank within the Department of Transfusion Medicine, Max Healthcare, New Delhi, India, between January 2010 and December 2024. Eligible donations were all blood donors, both voluntary and replacement, who were screened as per regulatory guidelines through established protocols, including informed consent, medical history, and physical examination, and for whom serology and NAT testing results were available. All the blood donations were collected from eligible donors following standard blood safety procedures and mandatory informed consent for blood sample testing through serology and NAT, including permission for HIV testing. Donations without NAT and/or serology results were excluded.

Serological testing and NAT

**Figure 1:**
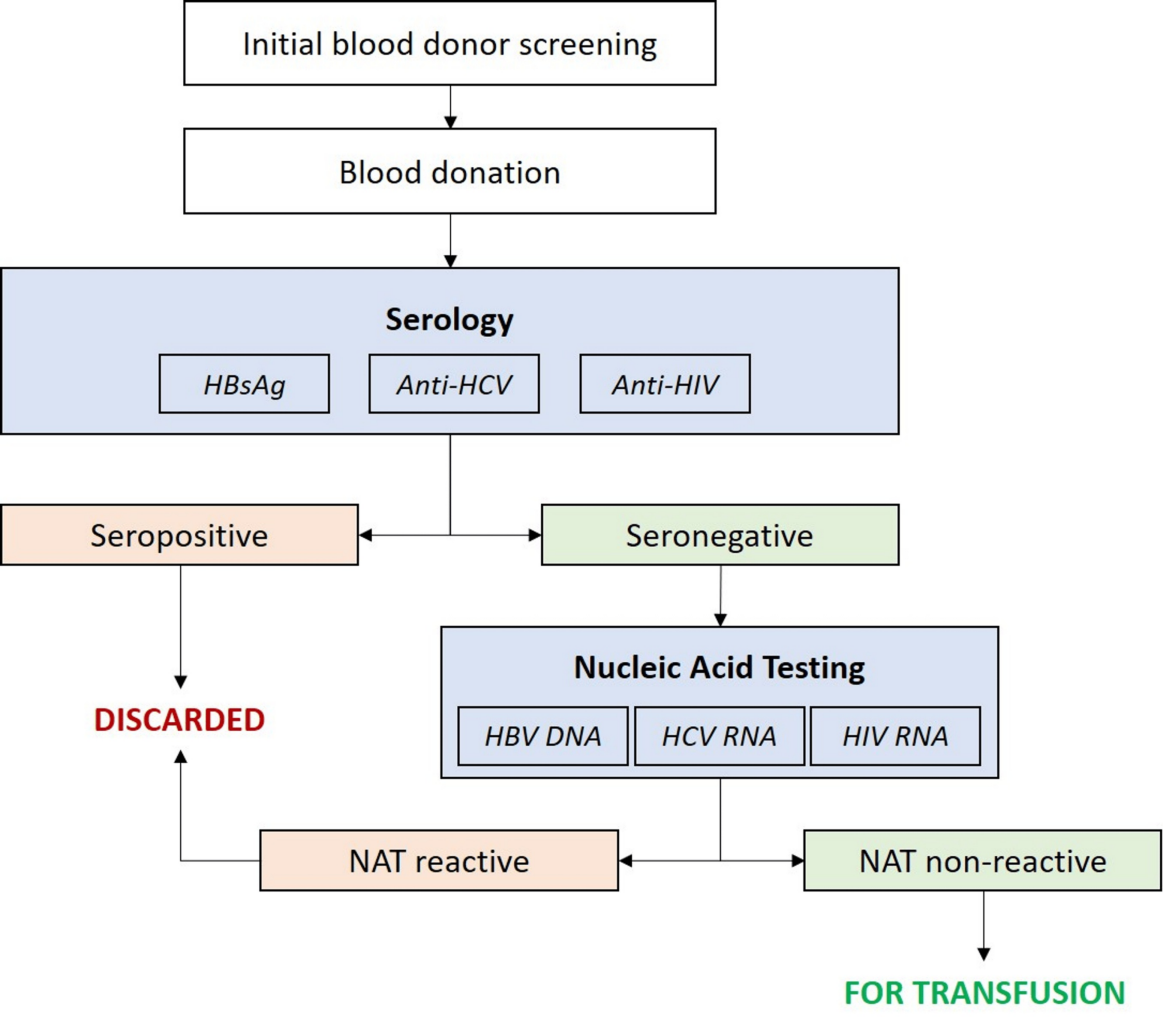
Serology and NAT methodology HBsAg: hepatitis B surface antigen; HCV: hepatitis C virus; HIV: human immunodeficiency virus; NAT: nucleic acid test; DNA: deoxyribonucleic acid; RNA: ribonucleic acid

After blood collection, all donations underwent a series of serological tests to detect TTIs, including HBV (via hepatitis B surface antigen (HBsAg)), HCV (via anti-HCV antibodies), and HIV (via anti-HIV-1 and anti-HIV-2 antibodies) (Figure 1). All serological tests were conducted through CLIA, according to the manufacturers' specifications. Identification and labelling of seroreactive samples were performed, adhering to the relevant NACO/NBTC guidelines at the time of serological testing. All seropositive donations were summarily discarded without further investigation.

Donations that were declared seronegative underwent NAT for the three viral TTIs (i.e., HBV, HCV, and HIV). For the purpose of this study, NAT was performed on minipools of 6 donations using polymerase chain reaction (PCR)-based MP-NAT via the Roche Cobas® TaqScreen MPX test v2.0 (Roche, Basel, Switzerland) on the fully automated s201 from 2010 to 2023 and the Cobas® 5800 system from 2024 onwards (Roche). This NAT platform can detect different strains and genetic variants of HBV (genotypes A to F), HCV (genotypes 1a, 1b, 2, 2a, 2b, 3a, 4, 4a, 4acd, 4d, 5a, 6, 6ab, and 6c), HIV-1 (including subtypes A, AE, AG, B, C, D, E, F, G, G-BG, BF, and J), HIV-2 (including subtypes A and B) [11]. Minipools of all NAT-reactive donations were resolved, and individual donations were subject to repeat NAT using the same platform to identify the seronegative donation harbouring the TTI. The identified donation was discarded despite the seronegative result. All seronegative donations with NAT reactivity were considered ‘NAT yield’ for the specific TTI.

The discarded seropositive and NAT-reactive samples were referred for counselling and further management as per established regulatory guidelines and clinical protocols.

Data handling, statistical analysis, and data availability

All data were entered electronically, and descriptive data analysis was performed using Microsoft Excel 2019 (Microsoft Corporation, Redmond, WA). Comparative analysis was not performed. The complete datasets used to reach the study conclusions can be obtained from the corresponding author upon reasonable request.

Ethics committee and informed consents

All blood donors were either replacement or voluntary donors, and their informed consent for blood sample testing through serology and NAT, including permission for HIV testing, was obtained following mandatory regulatory guidelines applicable at the time of blood donation. Since this study involved analysis of routinely collected anonymised data, ethics committee approval was determined not necessary.

## Results

Donor profile and serology

Between the 15-year period of January 2010 and December 2024, our blood bank received a total of 2,11,555 blood donations, of which 2,04,609 (96.72%) were from replacement/family donors, and the remaining 6,946 (3.28%) from voluntary donors.

A total of 3,333 (1.58%) donations were seropositive for viral TTIs, and most of these were from replacement donors (N=3,289; 98.68%) and from male donors (N=3,234; 97.03%). Seropositivity was most frequent for HBsAg (N=1,460; 43.78%), followed by anti-HCV (N=1,213; 36.39%) and HIV (N=660; 19.8%). In each of the three viral TTIs, seropositivity was higher amongst replacement donations than voluntary donations and amongst male donors compared to female donors (Table 1). This trend was observed in each calendar year of the data collection period (Tables 2-5).

**Table 1:** Seropositive results stratified based on gender, type of blood donation, and type of viral infection TTI: transfusion-transmitted infection; HBsAg: hepatitis B surface antigen; HCV: hepatitis C virus; HIV: human immunodeficiency virus

	Replacement donations (N=2,04,609)	Voluntary donations (N=6,946)	Total donations (N=2,11,555)	
Donations with seropositive results for viral TTIs (N, % of all donations)				
Total	3,289 (1.61%)	44 (0.63%)	3,333 (1.58%)	
Male	3,197 (1.56%)	37 (0.53%)	3,234 (1.53%)	
Female	92 (0.04%)	7 (0.10%)	99 (0.05%)	
Individual viral TTIs (N, % of all seropositive donations)				
HBsAg				
Total	1,440 (43.78%)	20 (45.45%)	1,460 (43.80%)	
Male	1,411 (44.14%)	19 (51.35%)	1,430 (44.22%)	
Female	29 (31.52%)	1 (14.29%)	30 (30.30%)	
Anti-HCV				
Total	1,199 (36.45%)	14 (31.82%)	1,213 (36.39%)	
Male	1,161 (36.32%)	10 (27.03%)	1,171 (36.21%)	
Female	38 (41.30%)	4 (57.14%)	42 (42.42%)	
HIV				
Total	650 (19.76%)	10 (22.73%)	660 (19.80%)	
Male	625 (19.55%)	8 (21.62%)	633 (19.57%)	
Female	25 (27.17%)	2 (28.57%)	27 (27.27%)	

**Table 2:** Year-wise split of donations seropositive for viral TTIs (HBV, HCV, and HIV) (2010 to 2024) TTI: transfusion-transmitted infection; HBsAg: hepatitis B surface antigen; HCV: hepatitis C virus; HIV: human immunodeficiency virus

Year	Total donors (N)	Replacement donors (N, %)	Voluntary donors (N, %)	Viral TTI seropositive							
				Total (N)	Replacement donors		Voluntary donors		Male donors (N, %)	Female donors (N, %)	
					Total, N (%)	Crude TTI positivity	Total, N (%)	Crude TTI positivity			
2010	10,196	9,894 (97.04%)	302 (2.96%)	186	184 (98.92%)	1.86%	2 (1.08%)	0.66%	178 (95.70%)	8 (4.30%)	
2011	10,114	9,802 (96.92%)	312 (3.08%)	201	199 (99.00%)	2.03%	2 (1.00%)	0.64%	191 (95.02%)	10 (4.98%)	
2012	12,537	12,152 (96.93%)	385 (3.07%)	221	221 (100.00%)	1.82%	0 (0.00%)	0.00%	215 (97.29%)	6 (2.71%)	
2013	12,701	11,375 (89.56%)	1,326 (10.44%)	208	208 (100.00%)	1.83%	0 (0.00%)	0.00%	202 (97.12%)	6 (2.88%)	
2014	12,824	12,518 (97.61%)	306 (2.39%)	204	203 (99.51%)	1.62%	1 (0.49%)	0.33%	195 (95.59%)	9 (4.41%)	
2015	11,485	11,157 (97.14%)	328 (2.86%)	182	181 (99.45%)	1.62%	1 (0.55%)	0.30%	173 (95.05%)	9 (4.95%)	
2016	12,431	12,097 (97.31%)	334 (2.69%)	195	195 (100.00%)	1.61%	0 (0.00%)	0.00%	189 (96.92%)	6 (3.08%)	
2017	13,893	13,333 (95.97%)	560 (4.03%)	207	202 (97.58%)	1.52%	5 (2.42%)	0.89%	201 (97.10%)	6 (2.90%)	
2018	13,624	12,957 (95.10%)	667 (4.90%)	200	195 (97.50%)	1.50%	5 (2.50%)	0.75%	197 (98.50%)	3 (1.50%)	
2019	14,038	13,603 (96.90%)	435 (3.10%)	222	216 (97.30%)	1.59%	6 (2.70%)	1.38%	213 (95.95%)	9 (4.05%)	
2020	12,434	12,193 (98.06%)	241 (1.94%)	200	199 (99.50%)	1.63%	1 (0.50%)	0.41%	198 (99.00%)	2 (1.00%)	
2021	17,860	17,624 (98.68%)	236 (1.32%)	288	284 (98.61%)	1.61%	4 (1.39%)	1.69%	285 (98.96%)	3 (1.04%)	
2022	19,461	18,951 (97.38%)	510 (2.62%)	270	264 (97.78%)	1.39%	6 (2.22%)	1.18%	262 (97.04%)	8 (2.96%)	
2023	19,476	18,958 (97.34%)	518 (2.66%)	265	258 (97.36%)	1.36%	7 (2.64%)	1.35%	259 (97.74%)	6 (2.26%)	
2024	18,481	17,995 (97.37%)	486 (2.63%)	284	280 (98.59%)	1.56%	4 (1.41%)	0.82%	276 (97.18%)	8 (2.82%)	
Total	2,11,555	2,04,609 (96.72%)	6,946 (3.28%)	3,333	3,289 (98.68%)	1.61%	44 (1.32%)	0.63%	3,234 (97.03%)	99 (2.97%)	

**Table 3:** Year-wise split of HBsAg seropositive donations (2010 to 2024) HBsAg: hepatitis B surface antigen

Year	HBsAg seropositive: total (N)	HBsAg seropositive: replacement donors (N, %)		HBsAg seropositive: voluntary donors (N, %)	
		Male	Female	Male	Female
2010	75	73 (97.33%)	1 (1.33%)	1 (1.33%)	0 (0.00%)
2011	102	95 (93.14%)	6 (5.88%)	1 (0.98%)	0 (0.00%)
2012	88	87 (98.86%)	1 (1.14%)	0 (0.00%)	0 (0.00%)
2013	100	100 (100.00%)	0 (0.00%)	0 (0.00%)	0 (0.00%)
2014	87	83 (95.40%)	4 (4.60%)	0 (0.00%)	0 (0.00%)
2015	98	96 (97.96%)	2 (2.04%)	0 (0.00%)	0 (0.00%)
2016	81	78 (96.30%)	3 (3.70%)	0 (0.00%)	0 (0.00%)
2017	80	77 (96.25%)	1 (1.25%)	2 (2.50%)	0 (0.00%)
2018	81	77 (95.06%)	0 (0.00%)	4 (4.94%)	0 (0.00%)
2019	105	101 (96.19%)	1 (0.95%)	3 (2.86%)	0 (0.00%)
2020	88	86 (97.73%)	1 (1.14%)	1 (1.14%)	0 (0.00%)
2021	124	120 (96.77%)	2 (1.61%)	2 (1.61%)	0 (0.00%)
2022	116	113 (97.41%)	2 (1.72%)	1 (0.86%)	0 (0.00%)
2023	107	102 (95.33%)	1 (0.93%)	3 (2.80%)	1 (0.93%)
2024	128	123 (96.09%)	4 (3.13%)	1 (0.78%)	0 (0.00%)
Total	1,460	1,411 (96.64%)	29 (1.99%)	19 (1.30%)	1 (0.07%)

**Table 4:** Year-wise split of anti-HCV seropositive donations (2010 to 2024) HCV: hepatitis C virus

Year	Anti-HCV seropositive: total (N)	Anti-HCV seropositive: replacement donors (N, %)		Anti-HCV seropositive: voluntary donors (N, %)	
		Male	Female	Male	Female
2010	78	72 (92.31%)	5 (6.41%)	1 (1.28%)	0 (0.00%)
2011	73	69 (94.52%)	3 (4.11%)	0 (0.00%)	1 (1.37%)
2012	96	92 (95.83%)	4 (4.17%)	0 (0.00%)	0 (0.00%)
2013	90	84 (93.33%)	6 (6.67%)	0 (0.00%)	0 (0.00%)
2014	78	75 (96.15%)	2 (2.56%)	1 (1.28%)	0 (0.00%)
2015	64	61 (95.31%)	3 (4.69%)	0 (0.00%)	0 (0.00%)
2016	87	85 (97.70%)	2 (2.30%)	0 (0.00%)	0 (0.00%)
2017	97	93 (95.88%)	2 (2.06%)	1 (1.03%)	1 (1.03%)
2018	75	73 (97.33%)	2 (2.67%)	0 (0.00%)	0 (0.00%)
2019	62	57 (91.94%)	3 (4.84%)	0 (0.00%)	2 (3.23%)
2020	76	75 (98.68%)	1 (1.32%)	0 (0.00%)	0 (0.00%)
2021	84	83 (98.81%)	0 (0.00%)	1 (1.19%)	0 (0.00%)
2022	64	61 (95.31%)	2 (3.13%)	1 (1.56%)	0 (0.00%)
2023	99	94 (94.95%)	2 (2.02%)	3 (3.03%)	0 (0.00%)
2024	90	87 (96.67%)	1 (1.11%)	2 (2.22%)	0 (0.00%)
Total	1,213	1,161 (95.71%)	38 (3.13%)	10 (10.82%)	4 (0.33%)

**Table 5:** Year-wise split of anti-HIV seropositive donations (2010 to 2024) HIV: human immunodeficiency virus

Year	Anti-HIV (1, 2) seropositive: total (N)	Anti-HIV (1, 2) seropositive: replacement donors (N, %)		Anti-HIV (1, 2) seropositive: voluntary donors (N, %)	
		Male	Female	Male	Female
2010	33	31 (93.94%)	2 (6.06%)	0 (0.00%)	0 (0.00%)
2011	26	26 (100.00%)	0 (0.00%)	0 (0.00%)	0 (0.00%)
2012	37	36 (97.30%)	1 (2.70%)	0 (0.00%)	0 (0.00%)
2013	18	18 (100.00%)	0 (0.00%)	0 (0.00%)	0 (0.00%)
2014	39	36 (92.31%)	3 (7.69%)	0 (0.00%)	0 (0.00%)
2015	20	16 (80.00%)	3 (15.00%)	0 (0.00%)	1 (5.00%)
2016	27	26 (96.30%)	1 (3.70%)	0 (0.00%)	0 (0.00%)
2017	30	27 (90.00%)	2 (6.67%)	1 (3.33%)	0 (0.00%)
2018	44	42 (95.45%)	1 (2.27%)	1 (2.27%)	0 (0.00%)
2019	55	51 (92.73%)	3 (5.45%)	1 (1.82%)	0 (0.00%)
2020	36	36 (100.00%)	0 (0.00%)	0 (0.00%)	0 (0.00%)
2021	80	78 (97.50%)	1 (1.25%)	1 (1.25%)	0 (0.00%)
2022	90	82 (91.11%)	4 (4.44%)	4 (4.44%)	0 (0.00%)
2023	59	57 (96.61%)	2 (3.39%)	0 (0.00%)	0 (0.00%)
2024	66	63 (95.45%)	2 (3.03%)	0 (0.00%)	1 (1.52%)
Total	660	625 (94.70%)	25 (3.79%)	8 (1.21%)	2 (0.30%)

NAT results

After discarding a total of 4,470 seropositive donations for viral and other TTIs, the remaining 2,07,085 seronegative samples were subject to NAT. The year-wise NAT yields are presented in Table 6. Of the 205 NAT yields (including one case of HBV and HCV coinfection) over 15 years, most (N=164, 79.76%) were HBV NAT yields, followed by HCV (N=35, 16.83%) and HIV (N=7, 3.41%). The overall NAT yield rate was 1:1005, with the NAT yield rate for HBV the highest (1:1262) and that for HIV the lowest (1:29,583). The NAT yields were the highest between the years 2014 and 2024, with a peak in 2016 (32 NAT yields).

**Table 6:** Year-wise NAT yields for the three viral TTIs *One case had HBV-HCV co-infection; #NAT yield rate was calculated based on the total samples that underwent NAT. NAT: nucleic acid testing; TTI: transfusion-transmitted infection; HBsAg: hepatitis B surface antigen; HCV: hepatitis C virus; HIV: human immunodeficiency virus

Year	HBV NAT positives, N(%)	HCV NAT positives, N(%)	HIV NAT positives, N(%)	Total NAT postiives, N
2010	5 (62.50%)	1 (12.50%)	2 (25.00%)	8
2011	2 (40.00%)	2 (40.00%)	1 (20.00%)	5
2012	2 (50.00%)	2 (50.00%)	0 (0.00%)	4
2013	6 (100.00%)	0 (0.00%)	0 (0.00%)	6
2014	13 (76.47%)*	5 (29.41%)*	0 (0.00%)	17*
2015	15 (93.75%)	0 (0.00%)	1 (6.25%)	16
2016	27 (84.38%)	5 (15.63%)	0 (0.00%)	32
2017	21 (95.45%)	1 (4.55%)	0 (0.00%)	22
2018	14 (87.50%)	2 (12.50%)	0 (0.00%)	16
2019	10 (83.33%)	1 (8.33%)	1 (8.33%)	12
2020	14 (100.00%)	0 (0.00%)	0 (0.00%)	14
2021	10 (76.92%)	3 (23.08%)	0 (0.00%)	13
2022	8 (100.00%)	0 (0.00%)	0 (0.00%)	8
2023	10 (66.67%)	5 (33.33%)	0 (0.00%)	15
2024	7 (41.18%)	8 (47.06%)	2 (11.76%)	17
Total (%)	164* (79.76%)	35* (16.83%)	7 (3.41%)	205*
NAT yield rate#	1:1262	1:5916	1:29,583	1:1005

## Discussion

The results of our study reinforce the significance of NAT in enhancing blood safety in India. Our 15-year data identified that replacement donations continue to drive most blood donations, despite our blood bank being located at a tertiary care centre in urban North India. Unsurprisingly, replacement donations in our study accounted for the dominant proportion of seropositive samples, which aligns with previous reports from India [6,12]. Despite the knowledge that stringent donor selection is the cornerstone for blood safety [13], it might not be practically feasible to ensure clean donors in such a replacement donor-dominant environment. Considering that each donated blood unit is typically separated into multiple components, a single seronegative donation harbouring latent infection could expose multiple recipients to TTIs. In the present study, we identified 205 latent infections through NAT that were missed by serology, including one case of HBV and HCV co-infection: this averages around 14 cases per year. This means NAT directly prevented at least 205 cases of TTI transmission. Assuming each donation was split into three components and transfused to different recipients, the potential number of individuals protected by NAT in our centre could be as high as 615. In this context, depending on serology alone to identify TTIs in India can compromise blood safety to a considerable extent.

Previous reports have observed that transfusion services in India suffer from fragmentation, inconsistent regulations, and poor adherence to quality standards, leading to significant variations in donor selection and TTI screening through serology across centres in the country. While urban blood banks use ELISA or CLIA assays of varying sensitivity and specificity for serology, semi-urban and rural centres often use rapid tests with questionable accuracy. These, and other factors such as inadequate quality assurance, lack of equipment calibration, and absence of validated results, highlight an urgent need for standardised protocols and stringent donor selection [14]. Enhancing awareness among blood donors is crucial to reverse the trend of replacement donor dominance in the pool of blood donors. However, until this can be achieved, a mechanism must be in place to identify latent TTIs that are missed by serology. We believe mandatory routine NAT testing can bridge this gap efficiently.

NAT enhances transfusion safety by detecting viral RNA/DNA before seroconversion, thereby significantly reducing the WP of detection of the viral TTIs. Among the two NAT formats, the sensitivity of ID-NAT is reported to be often higher than that of MP-NAT, largely because of the pooling dilution effect seen with the latter [8,15]. Despite this, MP-NAT remains the more cost-effective format globally, particularly in resource-limited settings such as India, because of its ability to screen multiple samples at the same time [8,16-18]. Earlier MP-NAT methods tested pools of up to 96 samples, but advancements have led to smaller pools of six to eight samples, thereby enhancing sensitivity while maintaining cost-effectiveness [8,15]. Thus, while high-income countries often use ID-NAT as the standard, MP-NAT is more suitable for developing nations like India, primarily due to challenges associated with costs and accessibility [10].

The pattern of seroreactivity and NAT yields observed in our study suggests that the seroprevalence of HIV is considerably lower than HBV and HCV, which indirectly suggests a better awareness among donors regarding HIV compared to HBV and HCV. Similar to previous reports, we observed that HBV infection was the most frequently picked up TTI by both serology and NAT [10,16]. The risk of HBV transmission because of occult HBV infection (OBI) not detected by HBsAg has been recognised as a major challenge in transfusion medicine [8,10]. This pattern of HBV driving serology and NAT yields is similar across developing countries, similar to ours [10]. The NAT yields observed in our study for the three viral TTIs are considerably higher than those from developed countries: in Croatia, for example, a 2017 study reported ID-NAT yields of 1: 36,900 for HBV, 1:15 million for HCV, and 1:1.5 million for HIV [8]. For Indian blood donations to achieve such safety standards, it is imperative that efforts are focused on both the promotion of voluntary blood donation as well as the regulation and possible government support for the implementation of routine NAT screening in blood banks across the country.

As of 2017, only around 2% of all blood banks in India routinely perform NAT, and these blood banks are mostly situated in larger cities and tertiary care centres, predominantly in North and South India [10]. The main drivers of the low acceptance of NAT in India are the perceived high cost associated with NAT, including the need for specialised equipment and trained personnel [1,19]. Nevertheless, we believe that there is a strong case for a push towards mandating routine NAT in India, regardless of the format (ID-NAT or MP-NAT). Each of the three viral TTIs (HBV, HCV, and HIV) results in chronic infections with prolonged hospitalisation that require continuous monitoring through a multitude of investigations, have expensive treatment courses, are associated with multiple complications including risk of progression to cancers, and have a major negative impact on the quality of life: consequently, preventing even a single case of these TTIs has the potential to yield meaningful public health and economic implications for both the patient and society. However, a formal cost-effectiveness analysis considering all costs and consequences is essential to quantify the extent of benefits vis-à-vis the investment required for routine NAT implementation in India.

The primary limitation of this study is its single-centre design, which might restrict generalisability across the diverse blood donor populations and screening practices in India. Further, though the study hints at the possible cost-effectiveness of NAT, a formal analysis of all costs and consequences was not performed. Despite these limitations, our study has shed light on the number of transfusions of seronegative samples harbouring latent infections that NAT was able to prevent over a period of 15 years in a single centre. This number would have been higher if NAT were adopted across the country, thereby preventing a much larger number of patients from acquiring TTIs. These results must nudge the regulators to realise the positive impact and long-term cost-effectiveness of NAT. With some states in India contemplating mandating NAT in blood banks [20], a nationwide push for mandatory NAT screening of blood donations is essential to enhance blood safety in India. To further support this concept, formal investigations of the cost-effectiveness of NAT testing in addition to serology in different settings across the country are required.

## Conclusions

Our study has shown that NAT prevented at least 205 TTIs missed by serology over 15 years in a real-world setting, thereby demonstrating its crucial role in blood safety. Consistent with previous studies, replacement donors constituted the majority of the donor pool and were also the primary source of seropositive donations, emphasising the need for greater donor awareness. HBV was the most frequently detected infection by both serology and NAT, highlighting the challenge of OBI in transfusions. Despite barriers relating to cost, infrastructure, and awareness, the long-term benefits of NAT in preventing chronic HBV, HCV, and HIV infections are likely to far outweigh its initial investment. Our research suggests that a nationwide mandatory NAT implementation is not only a clinical necessity but also an economic imperative. In addition to mandating NAT for blood screening, practical regulations aimed at enhancing blood safety and increasing donor awareness are essential to improve the standards and patient outcomes of transfusion in India. Future research should focus on formal cost-effectiveness analyses to drive policy decisions toward mandatory NAT screening.
